# Spatiotemporal dynamic and regional differences of public attention to vaccination: An empirical study in China

**DOI:** 10.1371/journal.pone.0312488

**Published:** 2024-12-23

**Authors:** Yaming Zhang, Xiaoyu Guo, Yanyuan Su

**Affiliations:** 1 School of Economics and Management, Yanshan University, Qinhuangdao, China; 2 Center for Internet Plus and Industry Development, Yanshan University, Qinhuangdao, China; Shanghai Maritime University, CHINA

## Abstract

**Background:**

Internet searches offer an indicator of public attention and possible demand for certain things. Studying the spatiotemporal characteristics of the public’s concern for vaccination can determine the spatiotemporal distribution of demand for vaccines in China, and capture the changes in the health awareness of the Chinese population, thus informing future vaccination strategies.

**Methods:**

Based on the collection of Baidu search indices for vaccination-related keywords in 363 cities in China, This paper seeks to explore the spatiotemporal changes and regional differences in public attention toward vaccination in China by using the seasonal index, seasonal concentration index, Herfindahl index, Moran index, and Dagum Gini coefficient.

**Results:**

The following findings are presented. First, there are significant seasonal fluctuations and unbalanced monthly distributions of vaccination-related public attention in China. Second, the public attention in Chinese cities shows the spatial characteristics of "leading in the east, followed by the central, western and northeastern regions". The spatial correlation of attention has been strengthened, and the high-high clusters are mainly distributed in the Beijing-Tianjin-Hebei (BTH), Yangtze River Delta (YRD), and Greater Bay Area (GBA) urban agglomerations. Third, regional differences in overall public attention narrowed in China, with intra-regional differences narrowing in seven regions (Northwest China, Central China, and so on), and intra-regional differences increasing in East China. The dominant role in the Gini coefficient changes from transvariation intensity to inter-regional differences.

**Conclusion:**

Major public health emergencies stimulate the public’s attention to health topics. Although the short-term increase in vaccination-related public attention was not observed to translate into a long-term increase in public vaccine literacy, the seasonal and regional differences in vaccination-related public attention in China have significantly narrowed before and after COVID-19, suggesting that the imbalance between public health literacy levels has improved.

## Introduction

Health literacy was considered to be a driver of health-promoting behaviors [1]. As an important element in improving public health literacy, increasing public acceptance of vaccination is a long-term task for the public health sector. In the unprecedented severity of the COVID-19 epidemic, vaccination, a highly cost-effective intervention in public health [2], played a huge role in responding to epidemics. However, the impact of COVID-19 on public vaccine literacy has not always been positive. On the one hand, routine vaccination has been hindered or canceled as COVID-19 has intensified [3]. On the other hand, public attitudes toward COVID-19 vaccination were ambiguous and uncertain [4–7], and vaccine hesitancy has become even more acute. The level of public attention toward vaccination has shown dramatic fluctuations with the epidemic [8–10]. Therefore, understanding changes in public attention toward vaccination before and after COVID-19 remains an important scientific question.

Changes in public attention are more timely than public opinion, which makes it a better measure of public interest in current events and trending topics [11, 12]. The social movements [13], public sentiment [14], and public mental health [12, 15–17] are all impacted by public attention. Using keywords related to vaccinations in internet searches shows how interested people are in vaccinations, reflects local vaccination needs and awareness, and serves as a foundation for international public health policy [18]. Studies on public attention toward vaccination can be broadly categorized into the following three groups.

The first type of research focused on exploring whether there is a correlation between public attention and vaccination behavior. While some scholars have found discrepancies between the Internet debates about vaccines and actual vaccination rates [19], more scholars have shown that online search behavior is associated with vaccination behavior [20, 21], and there is a positive correlation between vaccine-related attention and vaccination rate [22, 23]. The second type of research explored how search activities affect vaccination behavior. Worries about the disease [24] and scientific search content [25] can positively contribute to the correlation between public attention and vaccination behavior, while the proliferation of disinformation [26] can reduce vaccination rates. The first two types of studies revolve around the association between information-seeking behavior and vaccination behavior and have basically confirmed that there is indeed a significant correlation between the two. It can be seen that studying the popular attention to vaccination not only provides insights into their online information behavior but also indirectly into their real-life vaccination behavior. The third category analyzed the trends in public attention toward vaccination-related topics. For example, public attention to the 2015 measles outbreak in Berlin, Germany, was found to have a "Rubicon effect", with the closer the location of the outbreak, the higher the public attention [21]. In the global COVID-19 epidemic, the frequency of online searches related to COVID-19 vaccines by people in the United States [8, 27], China [10, 28], and Malaysia [29] showed a significant increase from 2020 to 2021 and fluctuated in response to the development of COVID-19.

COVID-19 is known to have long-lasting effects on the worldviews and health perceptions of individuals (both infected and uninfected) experiencing the epidemic [30, 31]. However, most of the existing studies have focused on the short-term impact of COVID-19 on public attention toward vaccination, ignoring the long-term effects. Major public health emergencies can undoubtedly strengthen public attention on health topics for a short time, but due to the highly variable nature of public attention, such intense interest may be difficult to sustain over a long period of time. In addition, there are regional differences in the public’s personalities [32], vaccination acceptance [33, 34], and access to health services [35], all of which may contribute to differences in public attention to vaccination. Therefore, it is worthwhile to continue to pay attention to the temporal evolution and regional differences of vaccination-related public attention in different regions before and after COVID-19.

In summary, utilizing Baidu search index data to investigate the Spatiotemporal characteristics and regional differences of vaccination-related public attention will allow for the novel detection of issues and aid in the long-term comprehension and management of public health behaviors. In this paper, we did the following based on the vaccination-related Baidu search index.

1. The public attention index (PAI) was constructed, and indicators such as the seasonal concentration index were used to depict the changes in public concern for vaccination.
2. The spatial and temporal characteristics of public attention were analyzed using measures such as the global Moran index and local Moran index.
3. Regional differences in public attention in 353 Chinese cities were presented based on the Daum Gini coefficient, and the sources leading to the differences were analyzed.

## Materials and methods

### Data sources

Research on public attention on the Internet mainly uses Google Trends [36], Baidu Index [11, 37], Sina Weibo [38, 39], Wikipedia article edits [40] or other indicators to evaluate the level of public interest in something. When the public is interested in a certain topic, they will frequently search online for terms related to the event, and thus the change in the number of web searches for related terms will reflect the degree of public attention to the corresponding event in real-time. As Baidu is the most commonly used search engine in China [41], the Baidu search index can be used to measure public attention [21, 42]. The Baidu search index is based on the number of searches made by netizens in Baidu, and it analyses and calculates the weighting of the frequency of searches for each keyword in Baidu’s web searches, which measures the degree of attention to keyword searches by Internet users and their continuous changes.

### Methods

**Fig 1:**
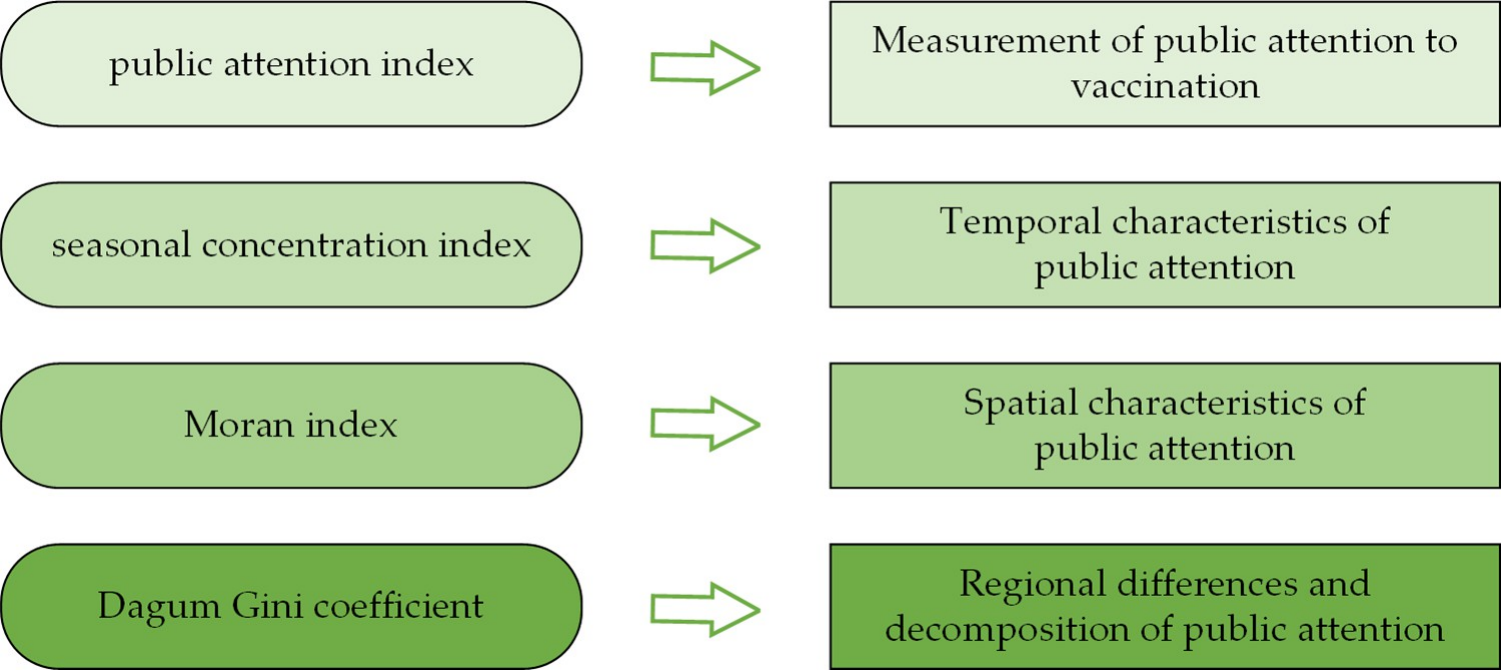
Research design for the spatiotemporal dynamics of public attention.

Based on the Baidu Index platform (https://index.baidu.com/), search data from 363 cities (356 prefecture-level cities, 4 municipalities, 2 special administrative regions, and Taiwan) were collected. Selecting "疫苗", "疫苗接种", "接种疫苗" and "打疫苗" as the relevant keywords of vaccination, the Baidu search indexes (BSI) of these four keywords were collected from 1 January 2019 to 31 December 2023. This paper described the spatiotemporal dynamics of the Chinese population’s vaccination attention based on Baidu search indexes (Fig 1).

The daily search indexes are summed up to obtain the public attention index (PAI) to vaccination on that day.


PAI=∑BSI (1)

The seasonal index (SI) is used as a time indicator and is calculated as follows:

$$SI=\frac{\mathrm{Average}\;\mathrm{for}\;\mathrm{the}\;\mathrm{same}\;\mathrm{month}\;\mathrm{of}\;\mathrm{each}\;\mathrm{year}}{\mathrm{Average}\;\mathrm{of}\;\mathrm{all}\;\mathrm{months}}\times 100\%$$ (2)

The seasonal concentration index (S) is used to explore the seasonal characteristics of public attention toward vaccination, and the formula for this index is as follows:

$$S=\sqrt{\sum_{i=1}^{12}{\left(M_{i}-8.33\right)}^{2}/12}$$ (3)

where *M*_*i*_ denotes the ratio of the monthly to annual total public attention index. The larger the value of S, the more uneven the distribution throughout the year and the greater the temporal variation in public attention.

The Herfindahl index (H) is a composite index that measures the degree of concentration, with values ranging from 0 to 1. The closer it is to 0, the lower the degree of agglomeration.

$$H=\sum_{i=1}^{n}P_{i}^{2}$$ (4)

where *P*_*i*_ is the ratio of the public attention index of a particular location to the total.

Moran index is used to measure the correlation characteristics of a variable’s spatial behavior, which generally includes global spatial autocorrelation and local spatial autocorrelation. The global Moran index reflects the spatial correlation between regions, and the results can determine whether or not clustering or outliers are occurring in space.

$$I=\frac{n\sum_{i=1}^{n}\sum_{j=1}^{n}\omega_{ij}(x_{i}-\bar{x})(x_{j}-\bar{x})}{(\sum_{i=1}^{n}\sum_{j=1}^{n}\omega_{ij})\sum_{i=1}^{n}{\left(x_{i}-\bar{x}\right)}^{2}}$$ (5)

where *x*_*i*_ and *x*_*j*_ are the indicator values of the variable *x* in cities *i* and *j*, respectively, and $\bar{x}$ is the mean value of *x*. *ω*_*ij*_ is the spatial matrix (we choose the adjacent spatial weight matrix), which is 1 when *x*_*i*_ and *x*_*j*_ are adjacent and 0 when they are not.

Further, the local Moran index can indicate where outliers or clusters occur. The "high-high cluster" indicates that areas of high attention are surrounded by areas of high attention, the "low-high outlier" indicates that areas of low attention are surrounded by areas of high attention, and so on.

The Dagum Gini coefficient is a method to decompose the Gini coefficient by subgroups, which can effectively measure the differences in the spatiotemporal distribution of the variable. The method effectively avoids the problems of crossover of sample data and discrepancies of regional sources. Drawing on the delineation of China’s human geography [43], this paper divides the 363 cities into eight regions, namely (Ⅰ) Northeastern China, (Ⅱ) Northern China, (Ⅲ) Northwestern China, (Ⅳ) Central China, (Ⅴ) Eastern China, (Ⅵ) Southern China, (Ⅶ) Southwestern China, and (Ⅷ) Qinghai-Tibet (S1 Table).

The Dagum Gini coefficient is calculated as:

$$G=\frac{\sum_{j=1}^{k}\sum_{h=1}^{k}\sum_{i=1}^{n_{j}}\sum_{r=1}^{n_{h}}\left|y_{ji}-y_{hr}\right|}{2n^{2}\bar{y}}$$ (6)

where *G* is the overall Gini coefficient, *n* is the number of cities, and *k* is the number of regions. *y*_*ji*_(*y*_*hr*_) is the public attention index of vaccination for each city within region *j*(*h*), and $\bar{y}$ is the average of public attention indices for each region. *n*_*j*_(*n*_*h*_) is the number of cities in region *j*(*h*), *i* and *r* are different cities in region *j*(*h*). *j* and *h* are different regions among the *k* regions, and *j* = 1,2,…,*k*.

The overall Dagum Gini coefficient (G) can be decomposed into contributions by three components: the intra-regional differences (G_w_), the inter-regional differences (G_nb_), and the intensity of transvariation (G_t_), and *G* = *G*_*w*_+*G*_*nb*_+*G*_*t*_ [44]. Where *G*_*w*_ denotes the gap in the distribution of public attention within region *j*(*h*), *G*_*nb*_ denotes the gap in the distribution of public attention between regions *j* and *h*, and *G*_*t*_ denotes the residuals for the cross-influence of public attention across regions. The calculation of each index is as follows.


$$\begin{array}{l} G_{w}=\sum_{j=1}^{k}G_{jj}p_{j}s_{j}\\ G_{jj}=\frac{\sum_{i=1}^{n_{j}}\sum_{r=1}^{n_{j}}\left|y_{ji}-y_{jr}\right|}{2n_{j}^{2}{\bar{y}}_{j}} \end{array}$$ (7)

$$\begin{array}{l} G_{nb}=\sum_{j=2}^{k}\sum_{h=1}^{j-1}G_{jh}(p_{j}s_{h}+p_{h}s_{j})D_{jh}\\ G_{jh}=\frac{\sum_{i=1}^{n_{j}}\sum_{r=1}^{n_{h}}\left|y_{ji}-y_{hr}\right|}{n_{j}n_{h}({\bar{y}}_{j}-{\bar{y}}_{h})} \end{array}$$ (8)

$$G_{t}=\sum_{j=2}^{k}\sum_{h=1}^{j=1}G_{jh}(p_{j}s_{h}+p_{h}s_{j})(1-D_{jh})$$ (9)

## Results

### Temporal analysis of the public attention to vaccination

**Fig 2:**
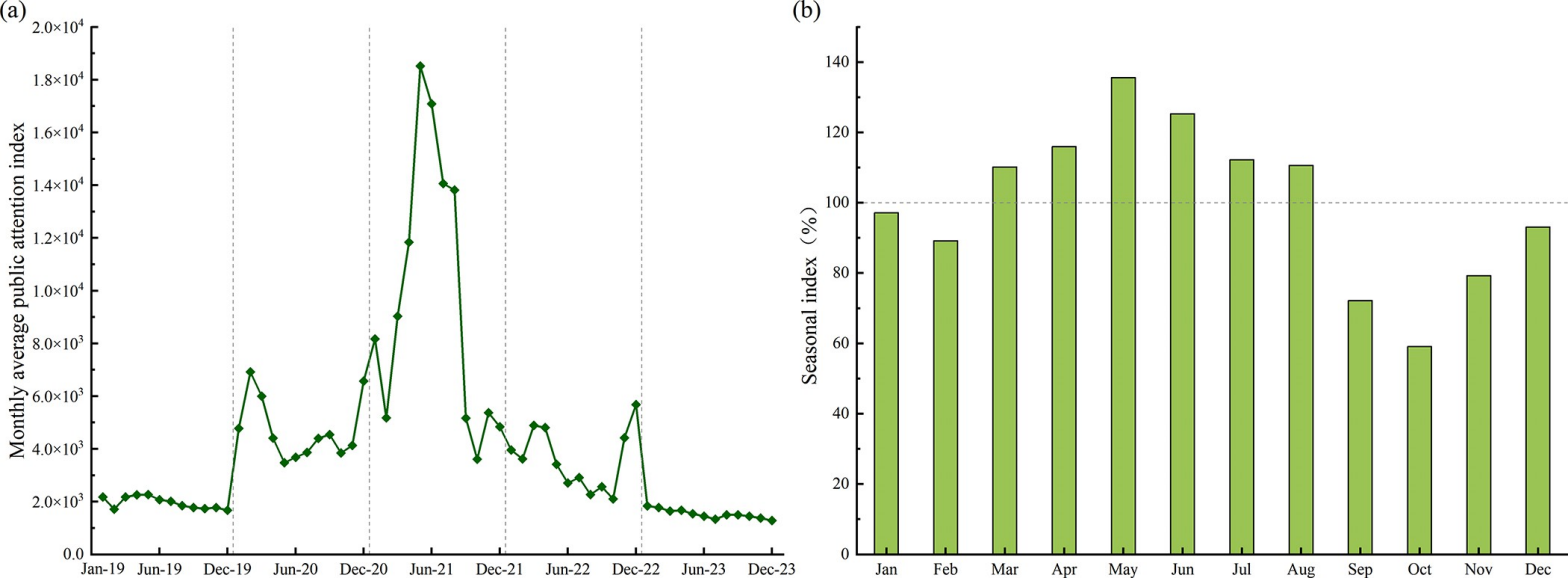
Time series characteristics of the public attention index in China. (a) The monthly average public attention index in China, 2019–2013. (b) The seasonal index of public attention index from January to December.

The monthly mean of PAI along with its seasonal index were calculated to reveal temporal trends in public attention (Fig 2). From Fig 2(A), it can be observed that the monthly average PAI curve as a whole has an inverted V-shape, showing that the Chinese public’s concern about vaccination has fluctuated over the observation period, first growing and then reducing.

There were substantial fluctuations in the average monthly PAI from 2020 to 2022 compared to 2019, indicating that COVID-19 had a non-negligible impact on public attention to vaccinations. The public’s expectations for the COVID-19 vaccine and concerns about its safety have led to a large number of Internet searches for vaccination-related information and a rapid increase in average monthly PAI. In 2021, along with the mass vaccination of the Chinese population with 2019-nCoVvaccine, the public’s attention to vaccination soared and the average monthly PAI peaked in May 2021 at 18526.26. Subsequently, public enthusiasm for vaccination-related topics declined, and the unusually high level of public attention to vaccination due to COVID-19 was largely finished by 2023. The level of public attention in 2023 is almost unchanged from that of 2019, with the average monthly PAI remaining between 1283.55 and 1834.32. It can be seen that the impact of the epidemic on the vaccination-related online attention of the Chinese public is mainly focused on the short-term impact, while the long-term impact and after-effects are weak.

The seasonal index (SI) was used to measure the monthly divergence characteristics of the public attention index. From Fig 2(B), it can be seen that the seasonal indices from March to August are all greater than 100%, while the seasonal indices from September to February are less than 100%. This indicates a seasonal fluctuation in PAI, with spring and summer being the peak seasons of public attention, while autumn and winter represent the off-seasons. According to the “Technical Guidelines for Influenza Vaccination in China (2023–2024)”, the public’s influenza vaccination volume and rate typically reach their peak between September and November each year. A clear “precursor effect” can be observed between public attention toward vaccination and vaccination behaviour. The public’s online information search behaviour was a precursor to vaccination behaviour, and vaccine recipients need to plan well in advance to understand the specific precautions of vaccination.

The results of the seasonal index indicate significant seasonal differences in the level of public attention toward vaccination. To explore the variation in seasonal differences across years, we calculated the seasonal concentration index (S) and the Herfindahl index (H) of public attention from 2019 to 2023, as shown in Table 1. The larger the value of the seasonal concentration index, the larger the seasonal difference in public attention, the more concentrated the distribution, and the more obvious the seasonal difference. It can be seen that seasonal differences in vaccination-related public attention are most significant in 2021, influenced by the COVID-19 vaccination process, whereas seasonal differences in public attention are not significant during non-COVID-19 epidemics. The Herfindahl index of 0.105634 in 2021 was higher than in other years, again indicating that public attention in that year was significantly influenced by the COVID-19 vaccination process and that the seasonal distribution of public attention was uneven.

**Table 1:** Seasonal concentration index and Herfindahl index of public attention index in China.

	2019	2020	2021	2022	2023
**Seasonal concentration index**	1.008963	1.897684	4.310895	2.586846	0.846031
**Herfindahl index**	0.084555	0.087655	0.105634	0.091363	0.084192

### Spatiotemporal distribution of the public attention to vaccination

**Fig 3:**
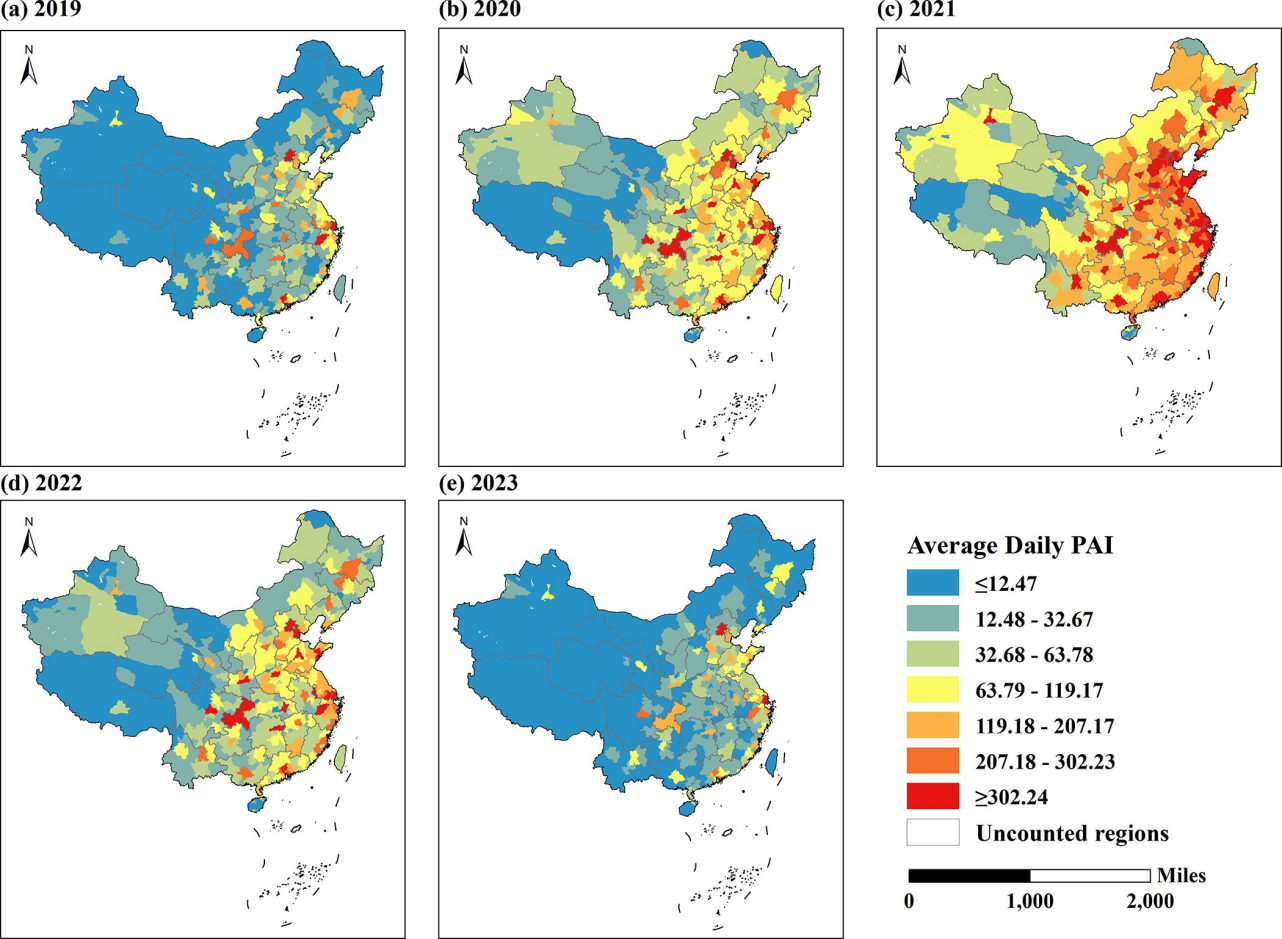
Average daily public attention index of 363 cities in China. Subfigures (a) to (e) depict the average daily PAI for 363 cities from 2019 to 2023, respectively. Note: Fig 3 was produced based on the standard map GS (2019) 1822 on the website of the Standard Map Service of the Ministry of Natural Resources of China, with no modifications to the boundaries of the base map.

To understand in more detail the changes in public attention toward vaccination before and after the COVID-19 outbreak in each city, the online search indices for vaccination-related keywords from 2019 to 2023 were obtained for 363 cities in China, and the average daily public attention indices were calculated for each city in each year. Using the natural breakpoint method provided by ArcGIS 10.8, the average daily PAI in 2019 was used to find the grouping breakpoints. The Natural Breaks method enables maximum similarity within each group and maximum dissimilarity between external groups, while the range and number of elements between each group are as similar as possible. The 363 cities in each year were divided into 7 groups respectively, and the results are shown in Fig 3.

The Hu Huanyong Line was widely recognized as the demarcation line for China’s population density distribution and economic and social patterns, and there was a clear difference in the distribution of population between the east and west sides of the line. Since the heat of public attention was closely related to the density of population distribution, the public attention in space also showed an obvious distribution characteristic of "high in the southeast and low in the northwest". As can be seen from Fig 3, although COVID-19 caused fluctuations in the public’s concern for vaccination, it had little effect on the spatial distribution of public attention, with "the south is greater than the north, and the east is greater than the west" both before and after the COVID-19.

The average daily public attention index of most cities increased significantly during the COVID-19 period, with 79% of cities having an average daily PAI of more than 63.79 in 2021. This suggests that popular interest in vaccination in most Chinese cities fluctuated considerably as a result of the COVID-19 epidemic. From Fig 3(b)-3(d), it can be seen that the impact of COVID-19 on public concern for vaccination varies significantly by region, with a greater impact on cities to the east and south of the Hu Huanyong line, where the fluctuation in public attention was more pronounced in cities of these regions.

In addition, COVID-19 has had less impact on public attention in cities with very low or very high levels of public attention, and the rank of average daily PAI has remained unchanged from 2019 to 2023 for these cities. In some cities, such as some cities in Hainan Province (Wuzhishan, Ding’an, Tunchang, Lingao, Baisha, Ledong, Baoting, and Qiongzhong), where the average daily PAI was below 12.48 in each of the past five years, and the level of public attention toward vaccination was extremely low even during the COVID-19 epidemic. In other cities, such as Beijing and Shanghai, the average daily public attention indexes for the past five years were greater than 302.23. Their large population bases and high levels of education have resulted in higher overall health literacy among city residents, and the public in these cities had a high level of attention toward vaccination-related health issues even before COVID-19.

Table 2 demonstrates the results of spatial autocorrelation of public attention to vaccination based on the global Moran index. The results show that the global Moran’s I of public attention is basically significantly positive at the 1% statistical level from 2019 to 2021, and the change in the value of the global Moran index gradually rises, indicating that the public attention to vaccination exhibits a positive spatial correlation characteristic of gradual enhancement. Subsequently, from 2021 to 2023, the value of global Moran’s I gradually declined, indicating that the positive spatial correlation of public attention gradually weakened.

**Table 2:** Spatial autocorrelation results for the public attention index in China.

	2019	2020	2021	2022	2023
**Global Moran’s I**	0.169284*	0.242644*	0.297982*	0.263270*	0.173933*
**Z-statistic**	5.189713	7.469090	9.073594	8.009005	5.357882

Note: * indicates a 1% significance level.

**Fig 4:**
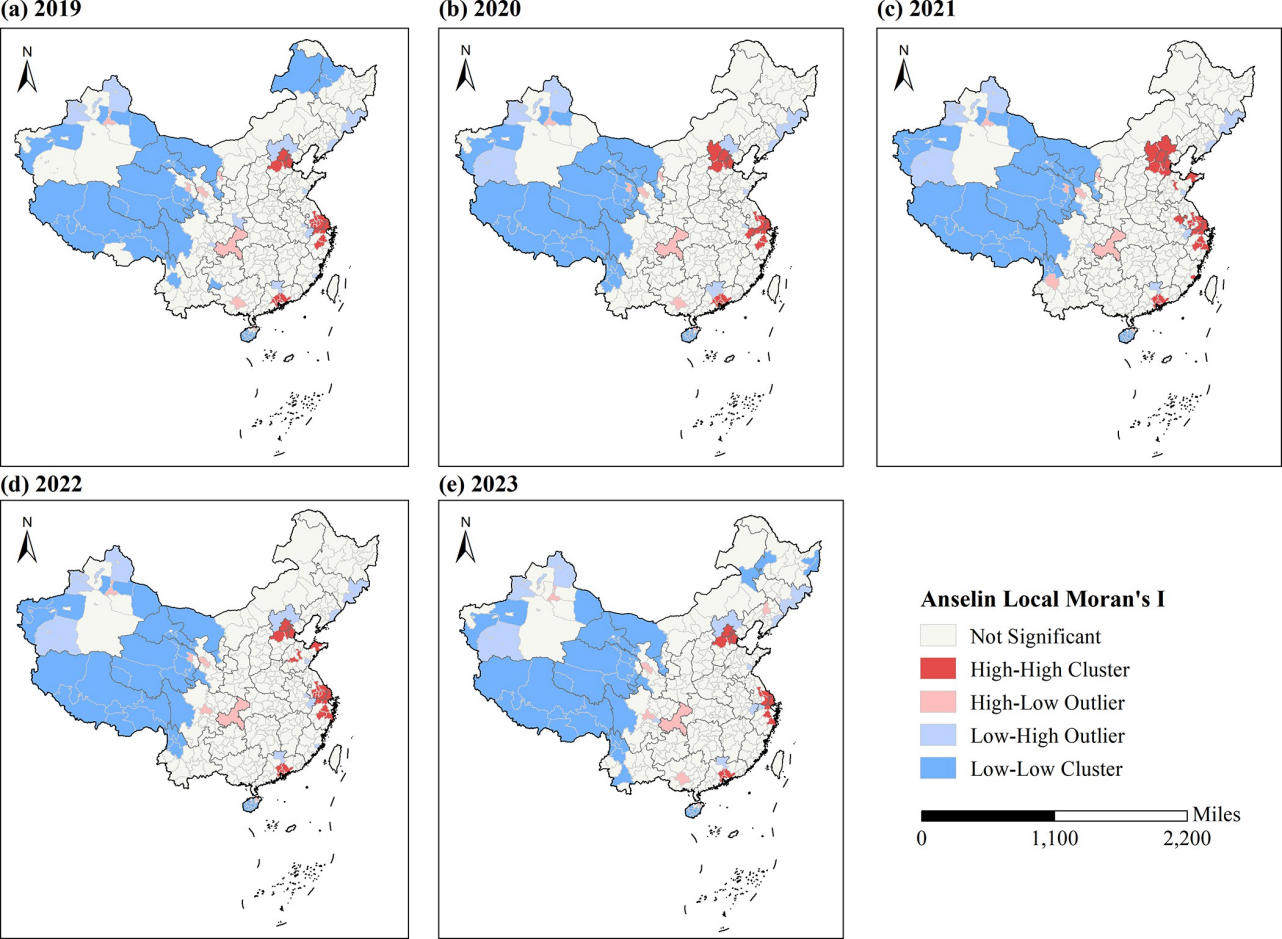
The cluster and outlier analysis of the average daily public attention index. Subfigures (a) to (e) depict the cluster and outlier analysis results from 2019 to 2023, respectively. Note: Fig 4 was produced based on the standard map GS (2019) 1822 on the website of the Standard Map Service of the Ministry of Natural Resources of China, with no modifications to the boundaries of the base map.

The cluster and outlier analyses were conducted using ArcGIS 10.8 based on Anselin Local Moran’s I on the average daily public attention indices of 363 Chinese cities per year to determine where significant clustering occurs (Fig 4). High-High Cluster indicates that the city itself and its neighbouring cities both have a high level of public attention, and High-Low Outlier indicates that the city itself has a high level of public attention but the public attention of its neighbouring cities is low.

As can be seen from Fig 4(A)–4(E), cities with high-high cluster patterns from 2019 to 2023 were concentrated in the Beijing-Tianjin-Hebei (BTH, Beijing, Tianjin, Baoding, Langfang), Yangtze River Delta (YRD, Shanghai, Suzhou, Nantong, Taizhou, Jiaxing, Shaoxing), and Greater Bay Area (GBA, Guangzhou, Huizhou, Dongguan, Zhongshan) urban agglomerations. The high-low outlier was formed in Haikou, Chongqing, Lanzhou, and Urumqi during 2019–2023. This shows that the cities with high levels of public interest were mainly concentrated in East and South China, while the vaccination-related public attention of the major provincial capital cities in Southwest and Northwest China was high, and the public attention of their surrounding cities was low.

The cities with low-low cluster patterns from 2019 to 2023 were largely concentrated in both Qinghai-Tibet and Northwest China. Most of these regions’ cities are sparsely populated, economically backward, and have low Internet penetration, resulting in a relatively low level of Internet-based public attention to vaccination. Hainan Province also forms a low-low clustering. Located in the southernmost part of China and surrounded by the sea on all sides, Hainan Province is a relatively independent geographic unit, while at the same time, there is a strong spatial correlation between the cities within Hainan Island. In addition, the low-low cluster was also formed in Xing’anmeng, Harbin, Qiqihar, and Hegang in Northeast China in 2023.

### Regional differences and decomposition of the public attention to vaccination

**Fig 5:**
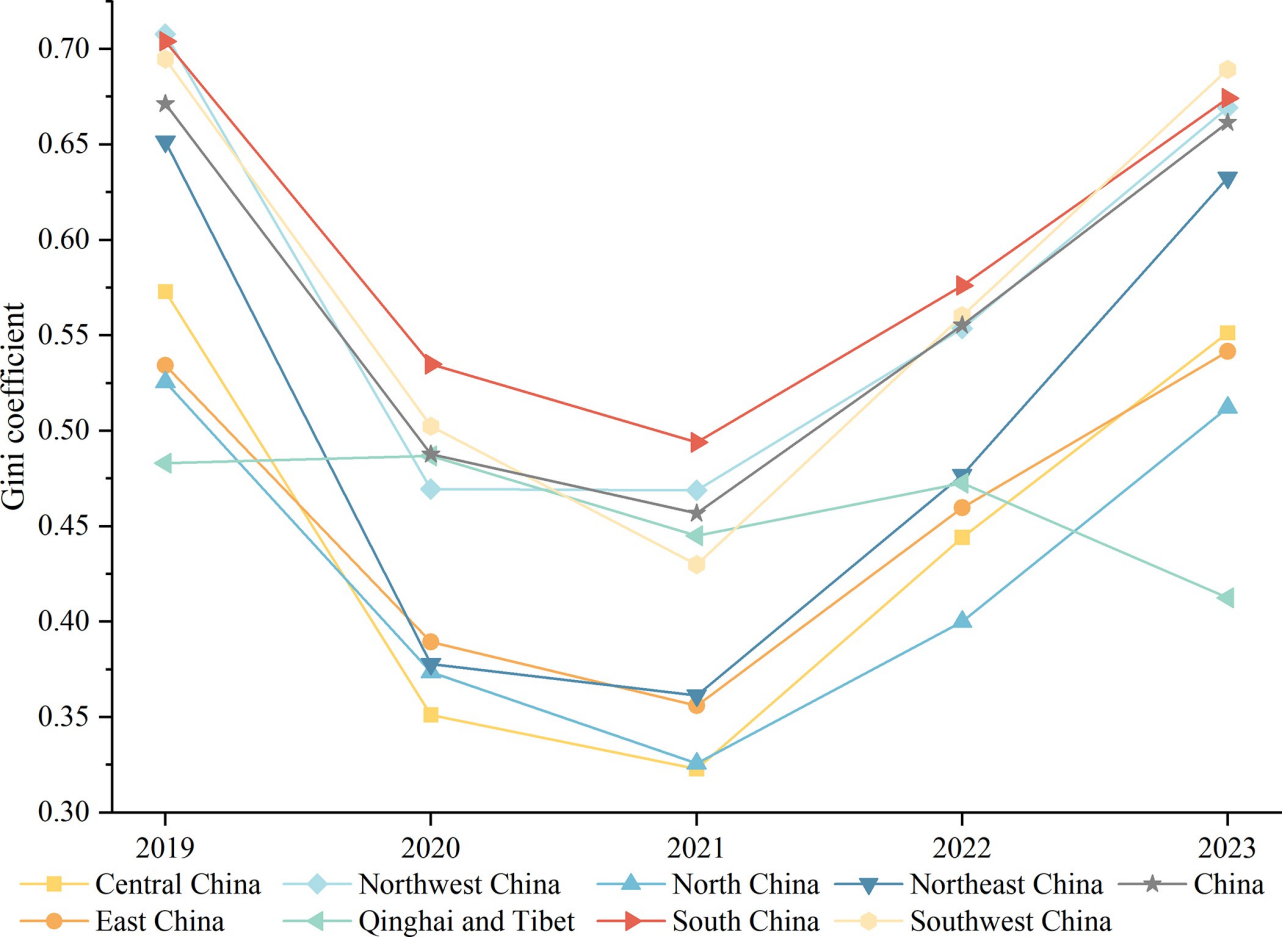
Tendency of the intra-regional Gini coefficient of public attention.

The Dagum Gini coefficient was used to characterize the spatiotemporal differences in public concern. The 363 cities were divided into 8 regions (S1 Table) and the Dagum Gini coefficient was measured from 2019 to 2023 based on the average daily public attention index for each city (Fig 5). The Gini coefficient of public attention in China ranges from 0.45677 to 0.67125, showing a decreasing and then increasing trend. Comparing before and after the COVID-19 outbreak, the national Gini coefficient decreased from 0.6725 in 2019 to 0.6615 in 2023, suggesting that COVID-19 has led to a reduction in the spatial variation in public attention. This may be related to a variety of factors such as the narrowing of the gap between regions in terms of Internet informatization, health literacy of the population, and healthcare resource allocation, as well as the long-tail effect brought about by the COVID-19 epidemic.

As can be seen in Fig 5, the fluctuating trend of the Gini coefficient in most regions is in line with the trend of the overall Gini coefficient, except for Qinghai-Tibet. Comparing the change in the Gini coefficient within the region before and after COVID-19, only East China rose, by 0.0074, while the remaining seven regions declined. This suggests that the gap in the level of public attention between cities within East China has become wider. Of the seven regions, Qinghai-Tibet had the largest decline of 0.07052, followed by Northwest China (0.03859) and South China (0.02990).

**Fig 6:**
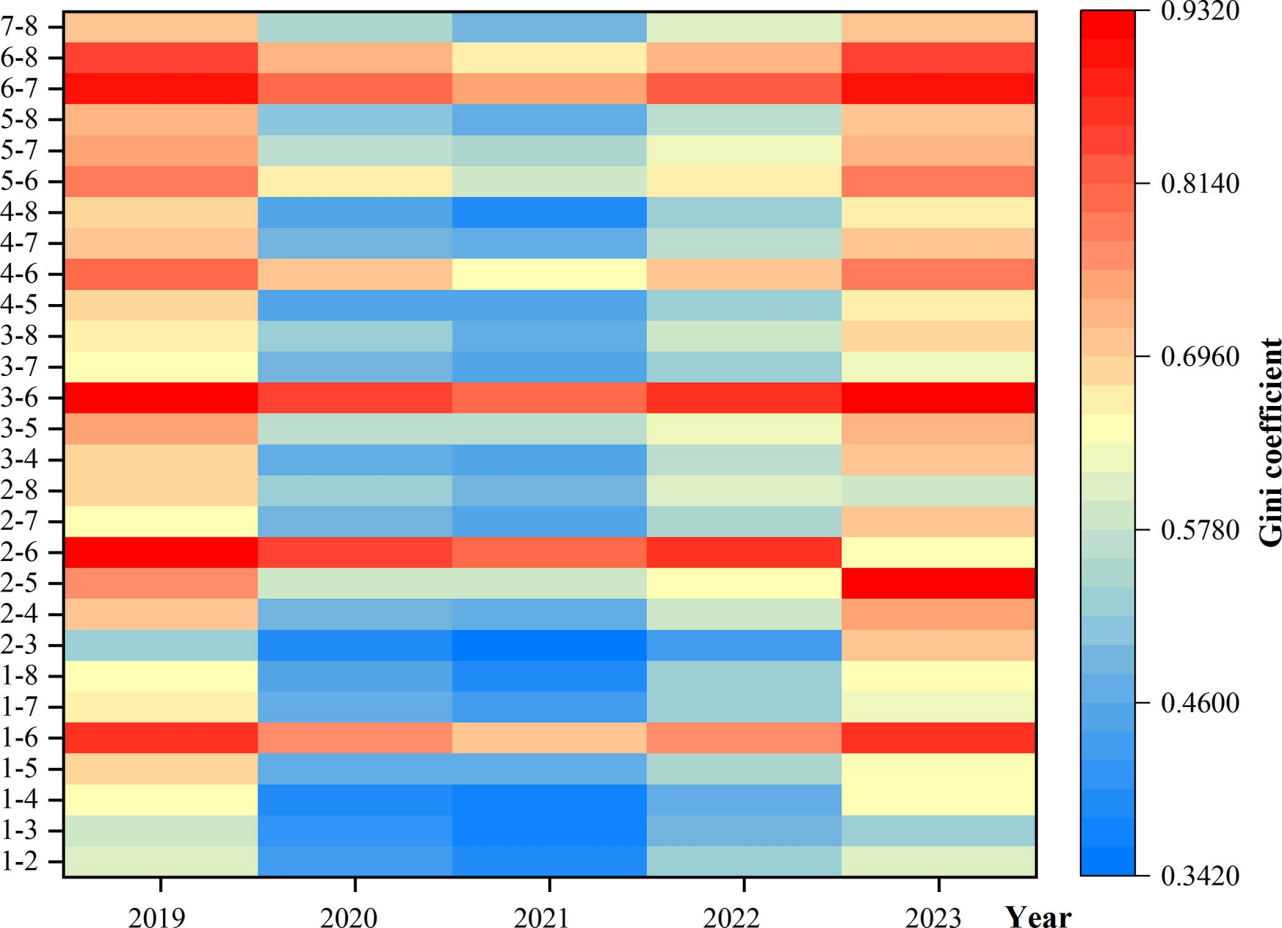
Tendency of the inter-regional Gini coefficient of public attention.

The heatmap was plotted (Fig 6) to visualize the trend of the inter-regional Gini coefficients more visually. As can be seen from Fig 6, the overall trend of inter-regional differences is "high on both sides and low in the middle", which means that the inter-regional Gini coefficient first decreased and then increased between 2019 and 2023. The fluctuation trend of the Gini coefficient of inter-regional differences is closely comparable with that of the overall Gini coefficient.

In terms of the changes before and after the COVID-19 (2019 vs. 2023), the inter-regional Gini coefficients of nine groups increased, namely 2–3 (0.1793), 2–5 (0.1718), 2–7 (0.0459), 2–4 (0.0284), 3–8 (0.0270), 3–4 (0.0250), 3–6 (0.0178), 1–6 (0.0080), and 6-7(0.0013). Among them, the increases of 2–3 and 2–5 were the most significant, which indicated that the imbalance of public attention between the two regions of East China and North China and Northwest China is becoming more and more obvious. Although regional differences in public attention have been reduced in the country as a whole, an imbalance in the development of public attention between China’s eastern and western regions still exists. East China has a high population density, education, and health literacy among its residents, and Internet penetration rate. After COVID-19, the population fully recognized the protective effect of vaccination on human health, and the frequency of proactive contact and knowledge related to vaccination increased, which may have contributed to the widening of the difference in the level of public attention between this region and other regions.

**Fig 7:**
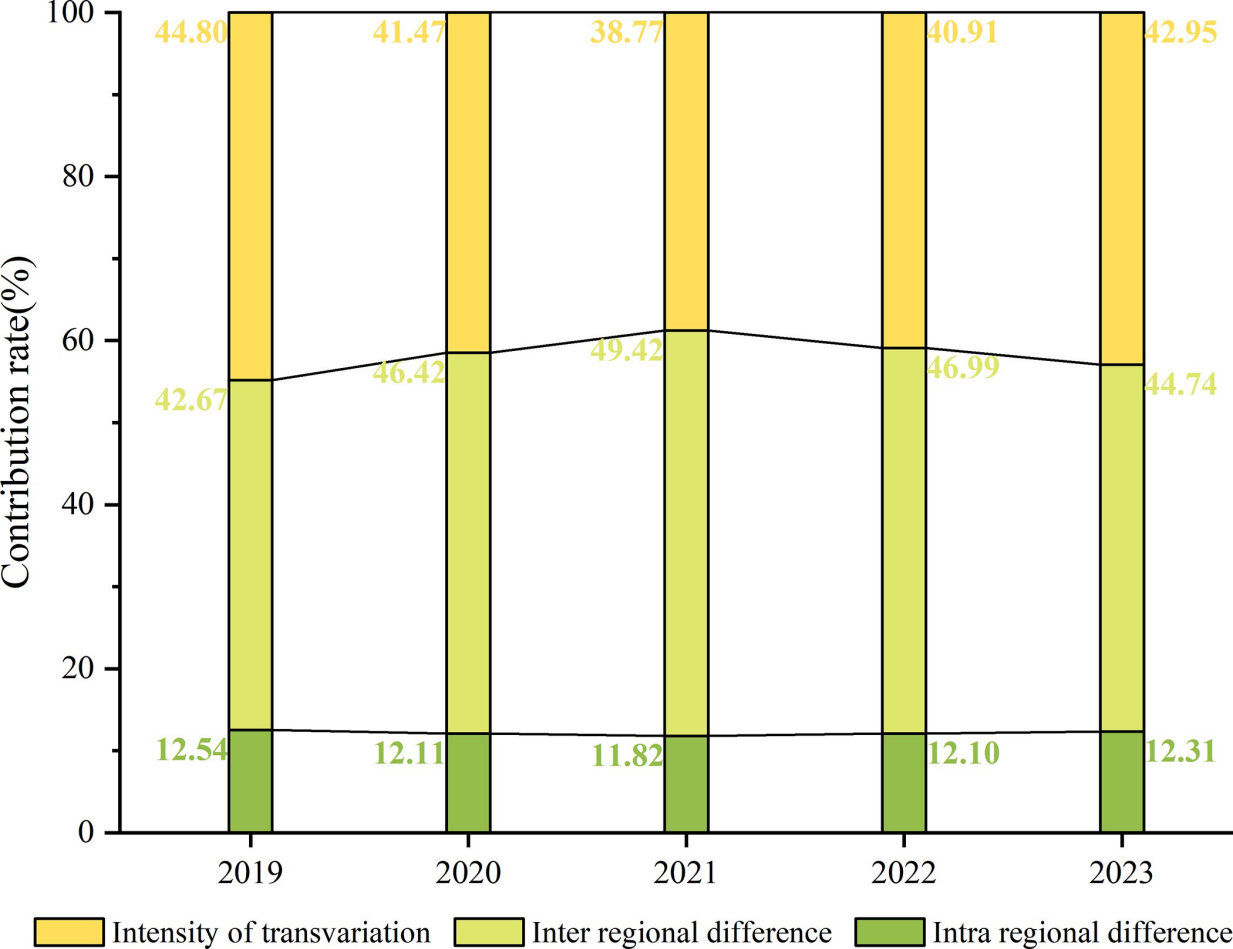
The sources of the differences in public attention and the trends of their contributions.

Fig 7 depicts the sources of the differences in public attention and the trends of their contribution rates. In terms of graphical features, the changes in the contribution rate of transvariation intensity (G_t_) and the inter-regional difference (G_nb_) are roughly symmetrically distributed. In terms of the evolution process, the trend of the contribution rate of intra-regional difference (G_w_) is more stable and has been low, while the contribution rate of inter-regional difference and transvariation intensity are more volatile. The change in contribution rates can be broadly divided into two phases. During the pre-COVID-19 period, the contribution rate of transvariation intensity was greatest. During the COVID-19 period and the post-COVID-19 period, inter-regional differences became the main cause of regional differences.

## Discussion

Major public health emergencies can undoubtedly strengthen public attention to topics in the health field for a short time, but due to the highly variable nature of public attention, this strong attention dissipates with the end of the epidemic. This led to our research questions: did COVID-19 have a long-term impact on public attention toward vaccination, and did COVID-19 exacerbate or mitigate differences in public attention between different regions of China? This paper focused on the spatiotemporal characteristics and changes of vaccination-related public attention in 363 cities in China during the five years before and after COVID-19 and explored the impact of major public health emergencies on vaccine literacy while describing the spatiotemporal changes in public vaccination attention.

Vaccination has long been the focus of the public sector, research organizations, and others, and the unprecedented severity of the COVID-19 outbreak has brought the topic to the attention of the general public. In particular, since the development and market promotion of the COVID-19 vaccine, the controversy over the safety of COVID-19 vaccination has been widely discussed on the Internet, and the general public’s interest in the topic of vaccination has gradually increased. According to the issue attention cycles theory, the occurrence of an event will inevitably trigger public attention to related topics, and it seems unsurprising that there was an increase in public attention to vaccination during the COVID-19 outbreak period. Interestingly, this study found that the public attention index of vaccination in China decreased by 432.55 after the end of COVID-19 compared to before COVID-19 occurred, and 73.76% of the cities had a smaller average daily PAI in 2019 than in 2023. It can be seen that COVID-19 only has a short-term "spotlight effect" on the public’s concern for vaccination, and this short-term effect can hardly be transformed into a long-term "echo effect" on the enhancement of the public’s vaccine literacy. In addition, the public may even become anxious and impatient because of the long duration of COVID-19, the repeated stimulation of related events, and the prolonged over-concentration of attention. The public’s reverse mentality was triggered, resulting in the "overrun effect", so the public’s interest in vaccination declined rapidly after the end of the COVID-19 outbreak, and was even lower than it was before the outbreak of the COVID-19 epidemic.

From 2019 to 2023, China’s public attention index showed an "inverted V" type of temporal change, with an increase followed by a decrease, and the real vaccination rate during this period also showed this characteristic. By 31 December 2021, the Chinese population had received a cumulative total of 2835 million doses of the COVID-19 vaccine, followed by a gradual decline in the net daily number of doses. Not only COVID-19 vaccinations but other routine vaccines have also been found to show an uptick in vaccination during COVID-19. For example, in the pneumococcal polysaccharide vaccination in Shanghai [45], and the influenza vaccination of primary and secondary school students in Beijing [46], a trend of increasing and then decreasing vaccination rates was found during the observation period. This may be related to factors such as public psychology and socio-cultural factors: the public attached extreme importance to the protection of their own health during the COVID-19 outbreak, and they were more eager than ever to achieve self-health protection through vaccination. The COVID-19 vaccine dominated the discussion of vaccines on social media platforms after the COVID-19 outbreak in 2020, and the discussion of other vaccines was overshadowed [19]. However, in China, the COVID-19 pandemic and COVID-19 vaccination campaigns did not significantly impede the process of other routine vaccines, and some routine vaccination rates instead declined after the end of COVID-19.

However, not all cities showed such a clear V-shaped time-series fluctuation in PAI. We found that the average daily PAI of 17 cities was below 12.48 in each of the past five years, i.e., the public attention toward vaccination in these cities has been very low. These 17 cities are located in Hainan Province (Wuzhishan, Ding’an, Tunchang, Lingao, Baisha, Ledong, Baoting, Qiongzhong), Qinghai Province (Haibei, Huangnan, Guoluo, Yushu), Xinjiang Uygur Autonomous Region (Alaer, Tumushuke, Wujiaqu), Hubei Province (Shennongjia Forestry District) and Tibet Autonomous Region(Ngari Prefecture). The size of the resident population and Internet users is the fundamental factor in determining the level of online attention [47]. Cities with higher population densities and larger Internet user bases have a higher level of public online attention. At the same time, it has been shown that any factor that may influence an individual’s need to access information, such as internet penetration [37], education level [48], and personal health literacy [49], can affect online attention. In addition, epidemics-related public attention in a city can be affected by spatial factors such as the geographical distance between the place and the source of the disease [21], the severity of the local epidemic [16, 18], and spatial analyses have shown that there are indeed spatial differences in the public attention in China, with a distribution pattern of "high in the south and east, and low in the north and west".

From 2019–2023, cities with high-high cluster patterns were largely concentrated in three urban agglomerations, namely, Beijing-Tianjin-Hebei (Beijing, Tianjin, Baoding, Langfang), Yangtze River Delta (Shanghai, Suzhou, Nantong, Taizhou, Jiaxing, Shaoxing), and Greater Bay Area (Guangzhou, Huizhou, Dongguan, Zhongshan). One possible explanation for the formation of high-high clusters could be that the city is subjected to similar external forces as its neighboring cities [50]. Relying on well-developed transport, communication, and other infrastructure networks, cities within the urban agglomerations have become increasingly connected in economic and cultural terms. Despite spatial differences between the core and peripheral areas of cities in terms of the operational cost of transportation [51] and so on, studies have shown that a variety of factors, such as information flows [52], population movements [53], and transport networks [54], have contributed to the convergence between cities within urban agglomerations.

Not only did public attention about vaccination change before and after the COVID-19 outbreak but so did regional differences in public attention. A comparison of the contribution of sources of differences in 2019 versus 2023 showed that the outbreak of COVID-19 has made inter-regional differences the main source leading to regional differences, accounting for 44.74%. However, the calculations in Fig 5 have found that the regional differences in public attention in China as a whole had narrowed, from which it can be inferred that the narrowing of the regional differences in public attention was mainly manifested in the narrowing of the differences between cities within a region, whereas the differences between cities belonging to different regions were increasing. It is interesting to note that Chinese cities are more and more clearly forming urban "communities", similar to city agglomerations and that the "boundaries" of these communities are becoming clearer, with stronger and more balanced connections within communities, and more and more sparse urban connections between communities.

This finding is particularly evident in the Qinghai-Tibet region. Of China’s eight geographic regions, the intra-regional Gini coefficient in Qinghai-Tibet declined the most, from 0.48301 in 2019 to 0.41249 in 2023. Although intra-regional differences have been mitigated in the Qinghai-Tibet region, the imbalance between Qinghai-Tibet and other regions, especially the coastal region, still exists. Calculations of the inter-regional Gini coefficients show that the Gini coefficients for 1–6 (Central China & Qinghai-Tibet), 2–6 (East China & Qinghai-Tibet), 3–6 (North China & Qinghai-Tibet), 4–6 (Northeast China & Qinghai-Tibet), 6–7 (Qinghai-Tibet & South China), and 6–8 (Qinghai-Tibet—Southwest China) have remained at high levels from 2019 to 2023. The reasons for this were closely related to factors such as population density and the level of Internet development, and the Qinghai-Tibet region remained vastly different from other regions in terms of economy and education.

## Conclusions

Focusing on the public’s health information search behaviour on the Internet, this paper analyses the changes in the Chinese public’s concern for vaccination before and after COVID-19 and explores the impact of COVID-19 on the public’s vaccine perceptions. At the same time, changes in regional differences in public attention are explored based on a geographical breakdown of the Chinese population. The conclusions of the study are set out below:

1. From the perspective of temporal changes in public attention in China, there are seasonal differences in public attention toward vaccination, most notably during the government-advocated mass COVID-19 vaccination period (2021). The peak season for public attention is from March to August.
2. From the perspective of spatial characteristics, the public attention in Chinese cities shows a clear spatial characteristic of "the east leads, followed by the central, western and northeastern regions". The public attention was characterized by positive spatial correlation from 2019 to 2023, and the spatial correlation effect was enhanced in the post-COVID-19 period (2023) compared with the pre-COVID-19 period (2019). At the same time, the high-high clusters have been formed within the Beijing-Tianjin-Hebei (BTH), Yangtze River Delta (YRD), and Greater Bay Area (GBA) urban agglomerations.
3. In terms of spatial differences in public attention, the Gini coefficient of public attention in China ranges from 0.45677 to 0.67125, with a decreasing and then increasing trend. The inter-regional differences were the main factor causing the regional differences in China’s public attention towards vaccination in 2023, followed by the intensity of transvariation, with the intra-regional differences contributing the least.

The analyses in this paper contribute to an understanding of Chinese popular health consciousness. Careful analyses of vaccination-related searches can help health authorities grasp the changing trends in public health perceptions and inform vaccination strategies and future efforts to protect public health. This study is therefore of significance in understanding the public’s health literacy, paying attention to public health behaviors, guiding public health work and even grasping the historical process of vaccination.

## Supporting information

S1 Table Administrative scopes of Chinese human geography regions. (DOCX)

## References

[1] Kim K, Shin S, Kim S, Lee EN. The Relation Between eHealth Literacy and Health-Related Behaviors: Systematic Review and Meta-analysis. J Med Internet Res. 2023;25:e40778. doi: 10.2196/40778 WOS:001009078900003. 36716080 PMC9926349

[2] Ozawa S, Schuh HB, Nakamura T, Yemeke TT, Lee YFA, Macdonald NE. How to increase and maintain high immunization coverage: Vaccination Demand Resilience (VDR) framework. Vaccine. 2023;41(45):6710–8. doi: 10.1016/j.vaccine.2023.09.027 WOS:001101511500001. 37798209

[3] Chakrabarti A, Bair EF, Thirumurthy H. Routine child immunizations in India during the COVID-19 pandemic. Ssm-Popul Hlth. 2023;22:101383. doi: 10.1016/j.ssmph.2023.101383 WOS:001042996400001. 36974277 PMC10014501

[4] Omer SB, Benjamin RM, Brewer NT, Buttenheim AM, Callaghan T, Caplan A, et al. Promoting COVID-19 vaccine acceptance: recommendations from the Commission on Vaccine Refusal, Acceptance, and Demand in the USA. Lancet. 2021;398(10317):2186–92. doi: 10.1016/S0140-6736(21)02507-1 WOS:000729467800016. 34793741 PMC8592561

[5] Islam MS, Siddique A, Akter R, Tasnim R, Sujan MSH, Ward PR, et al. Knowledge, attitudes and perceptions towards COVID-19 vaccinations: a cross-sectional community survey in Bangladesh. Bmc Public Health. 2021;21(1):1851. doi: 10.1186/s12889-021-11880-9 WOS:000707009300004. 34645399 PMC8513387

[6] Pogue K, Jensen JL, Stancil CK, Ferguson DG, Hughes SJ, Mello EJ, et al. Influences on Attitudes Regarding Potential COVID-19 Vaccination in the United States. Vaccines-Basel. 2020;8(4):582. doi: 10.3390/vaccines8040582 WOS:000601782000001. 33022917 PMC7711655

[7] Bianco A, Della Polla G, Angelillo S, Pelullo CP, Licata F, Angelillo IF. Parental COVID-19 vaccine hesitancy: a cross-sectional survey in Italy. Expert Rev Vaccines. 2022;21(4):541–7. doi: 10.1080/14760584.2022.2023013 WOS:000737033600001. 34949136

[8] Berning P, Huang L, Razavi AC, Boakye E, Osuji N, Stokes AC, et al. Association of Online Search Trends With Vaccination in the United States: June 2020 Through May 2021. Front Immunol. 2022;13:884211. doi: 10.3389/fimmu.2022.884211 WOS:000810776600001. 35514956 PMC9066639

[9] Maugeri A, Barchitta M, Agodi A. Using Google Trends to Predict COVID-19 Vaccinations and Monitor Search Behaviours about Vaccines: A Retrospective Analysis of Italian Data. Vaccines-Basel. 2022;10(1):119. doi: 10.3390/vaccines10010119 WOS:000748231100001. 35062780 PMC8778420

[10] Jiang LS, Ma QX, Wei SZ, Che GW. Online Public Attention of COVID-19 Vaccination in Mainland China. Digit Health. 2022;8:20552076211070454. doi: 10.1177/20552076211070454 WOS:000748559800001. 35096408 PMC8796085

[11] Huang YK, Xu XP, Su SN. Diverging from News Media: An Exploratory Study on the Changing Dynamics between Media and Public Attention on Cancer in China from 2011–2020. Int J Env Res Pub He. 2021;18(16):8577. doi: 10.3390/ijerph18168577 WOS:000689098300001. 34444326 PMC8391632

[12] Zhao YX, Cheng SX, Yu XY, Xu HL. Chinese Public’s Attention to the COVID-19 Epidemic on Social Media: Observational Descriptive Study. J Med Internet Res. 2020;22(5):e18825. doi: 10.2196/18825 WOS:000530021200001. 32314976 PMC7199804

[13] Hunt K, Gruszczynski M. The influence of new and traditional media coverage on public attention to social movements: the case of the Dakota Access Pipeline protests. Inform Commun Soc. 2021;24(7):1024–40. doi: 10.1080/1369118X.2019.1670228 WOS:000487587700001.

[14] Peng TQ, Sun GD, Wu YC. Interplay between Public Attention and Public Emotion toward Multiple Social Issues on Twitter. Plos One. 2017;12(1):e0167896. doi: 10.1371/journal.pone.0167896 WOS:000391949500003. 28081117 PMC5231282

[15] Liu MY, Luo XW, Lu WZ. Public perceptions of environmental, social, and governance (ESG) based on social media data: Evidence from China. J Clean Prod. 2023;387:135840. doi: 10.1016/j.jclepro.2022.135840 WOS:000925288700001.

[16] Hou KK, Hou TT, Cai LL. Public attention about COVID-19 on social media: An investigation based on data mining and text analysis. Pers Indiv Differ. 2021;175:110701. doi: 10.1016/j.paid.2021.110701 WOS:000628819000021. 33536695 PMC7843112

[17] Song C, Yin H, Shi X, Xie MY, Yang SJ, Zhou JM, et al. Spatiotemporal disparities in regional public risk perception of COVID-19 using Bayesian Spatiotemporally Varying Coefficients (STVC) series models across Chinese cities. Int J Disast Risk Re. 2022;77:103078. doi: 10.1016/j.ijdrr.2022.103078 WOS:000815707900003. 35664453 PMC9148270

[18] Hu F, Qiu LP, Xia W, Liu CF, Xi X, Zhao S, et al. Spatiotemporal evolution of online attention to vaccines since 2011: An empirical study in China. Front Public Health. 2022;10. ARTN 949482 doi: 10.3389/fpubh.2022.949482 WOS:000838352200001. 35958849 PMC9360794

[19] Chen QQ, Croitoru A, Crooks A. A comparison between online social media discussions and vaccination rates: A tale of four vaccines. Digit Health. 2023;9:20552076231155682. doi: 10.1177/20552076231155682 WOS:000928750100001. 36776405 PMC9912564

[20] Arendt F, Scherr S. Investigating an Issue-Attention-Action Cycle: A Case Study on the Chronology of Media Attention, Public Attention, and Actual Vaccination Behavior during the 2019 Measles Outbreak in Austria. J Health Commun. 2019;24(7–8):654–62. doi: 10.1080/10810730.2019.1652709 WOS:000482288200001. 31423919

[21] Arendt F, Scherr S. News-stimulated public-attention dynamics and vaccination coverage during a measles outbreak: An observational study. Soc Sci Med. 2020;265:113495. doi: 10.1016/j.socscimed.2020.113495 WOS:000607303000025. 33162194

[22] Uehara M, Fujita S, Shimizu N, Liew K, Wakamiya S, Aramaki E. Measuring concerns about the COVID-19 vaccine among Japanese internet users through search queries. Sci Rep-Uk. 2022;12(1):15037. doi: 10.1038/s41598-022-18307-4 WOS:000849490400001. 36057657 PMC9440921

[23] Moussa OZ, Takeuchi K. Does searching online for vaccination information affect vaccination coverage? Evidence from Sub-Saharan African countries. Economics & Human Biology. 2022;47:101181. doi: 10.1016/j.ehb.2022.101181 36116175

[24] Li L, Bautista OROE. Elaboration, Cancer Worry, and Risk Perception Mediate the Association Between News Attention on the Internet and Intention to Uptake HPV Vaccination: Extending the Cognitive Mediation Model. Int J Commun-Us. 2021;15:4862–83. WOS:000729944300152.

[25] Olagoke AA, Floyd B, Adebayo CT, Owoyemi A, Hughes AM. The Content of COVID-19 Information Searches and Vaccination Intention: An Implication for Risk Communication. Disaster Med Public. 2022;17:PII S1935789322002579. doi: 10.1017/dmp.2022.257 WOS:000906869400001. 36325832 PMC9837418

[26] Aquino F, Donzelli G, De Franco E, Privitera G, Lopalco PL, Carducci A. The web and public confidence in MMR vaccination in Italy. Vaccine. 2017;35(35):4494–8. doi: 10.1016/j.vaccine.2017.07.029 WOS:000410017500003. 28736200

[27] An L, Russell DM, Mihalcea R, Bacon E, Huffman S, Resnicow K. Online Search Behavior Related to COVID-19 Vaccines: Infodemiology Study. JMIR Infodemiology. 2021;1(1):e32127. doi: 10.2196/32127 34841200 PMC8601025

[28] Ma J, Luo JC, Xu MQ. An online longitudinal study about search index reflexing public attention of vaccinate against COVID-19. Ann Transl Med. 2022;10(15):827. doi: 10.21037/atm-22-3064 WOS:000826278800001. 36035006 PMC9403913

[29] Kow RY, Mohamad Rafiai N, Ahmad Alwi AA, Low CL, Ahmad MW, Zakaria Z, et al. COVID-19 Infodemiology: Association Between Google Search and Vaccination in Malaysian Population. Cureus Journal of Medical Science. 2022;14(9):e29515. doi: 10.7759/cureus.29515 36299936 PMC9588419

[30] Tan H, Peng SL, Zhu CP, You Z, Miao MC, Kuai SG. Long-term Effects of the COVID-19 Pandemic on Public Sentiments in Mainland China: Sentiment Analysis of Social Media Posts. J Med Internet Res. 2021;23(8):e29150. doi: 10.2196/29150 WOS:000685097800002. 34280118 PMC8360336

[31] Barzilay R, Moore TM, Greenberg DM, DiDomenico GE, Brown LA, White LK, et al. Resilience, COVID-19-related stress, anxiety and depression during the pandemic in a large population enriched for healthcare providers. Transl Psychiat. 2020;10(1):291. doi: 10.1038/s41398-020-00982-4 WOS:000568381800001. 32820171 PMC7439246

[32] Rentfrow PJ. Statewide Differences in Personality: Toward a psychological geography of the United States. Am Psychol. 2010;65(6):548–58. doi: 10.1037/a0018194 WOS:000281832800002. 20822196

[33] Renzi E, Baccolini V, Migliara G, Bellotta C, Ceparano M, Donia P, et al. Mapping the Prevalence of COVID-19 Vaccine Acceptance at the Global and Regional Level: A Systematic Review and Meta-Analysis. Vaccines-Basel. 2022;10(9):1488. doi: 10.3390/vaccines10091488 WOS:000858918800001. 36146566 PMC9506365

[34] Abedin M, Islam MA, Rahman FN, Reza HM, Hossain MZ, Hossain MA, et al. Willingness to vaccinate against COVID-19 among Bangladeshi adults: Understanding the strategies to optimize vaccination coverage. Plos One. 2021;16(4):e0250495. doi: 10.1371/journal.pone.0250495 WOS:000665456800020. 33905442 PMC8078802

[35] Guan M. Associations Between Geodemographic Factors and Access to Public Health Services Among Chinese Floating Population. Front Public Health. 2020;8:563180. doi: 10.3389/fpubh.2020.563180 WOS:000601284900001. 33363076 PMC7758272

[36] Lo KL, Liu HZ, Yang MH, Mi JJ. The relationship between public attention and COVID-19: evidence from the big data analysis of Google trends. Appl Econ Lett. 2022;29(17):1586–93. doi: 10.1080/13504851.2021.1948958 WOS:000668449500001.

[37] Ji Q, Yang JP, He QS, Chen HJ, Wang XR, Tang F, et al. Understanding Public Attention towards the Beautiful Village Initiative in China and Exploring the Influencing Factors: An Empirical Analysis Based on the Baidu Index. Land-Basel. 2021;10(11):1169. doi: 10.3390/Land10111169 WOS:000725798000001.

[38] Wang B, Jiang ZX, Cheng DW, Wang Z. Exploring public attention and sentiment toward carbon neutrality: evidence from Chinese social media Sina Weibo. Front Psychol. 2023;14:1200824. doi: 10.3389/fpsyg.2023.1200824 WOS:001012482600001. 37359875 PMC10285706

[39] Xu Z, Yen NY, Zhang H, Wei X, Lv ZH, Choo KKR, et al. Social Sensors Based Online Attention Computing of Public Safety Events. Ieee T Emerg Top Com. 2017;5(3):403–11. doi: 10.1109/Tetc.2017.2684819 WOS:000409342600010.

[40] Bennett WL, Segerberg A, Yang YK. The Strength of Peripheral Networks: Negotiating Attention and Meaning in Complex Media Ecologies. J Commun. 2018;68(4):659–84. doi: 10.1093/joc/jqy032 WOS:000449503700005.

[41] Jiang BZ, Zhu HJ, Zhang JH, Yan C, Shen R. Investor Sentiment and Stock Returns During the COVID-19 Pandemic. Front Psychol. 2021;12:708537. doi: 10.3389/fpsyg.2021.708537 WOS:000680385500001. 34354650 PMC8329237

[42] Arendt F, Scherr S. The Impact of a Highly Publicized Celebrity Suicide on Suicide-Related Online Information Seeking. Crisis. 2017;38(3):207–9. doi: 10.1027/0227-5910/a000455 WOS:000404128700009. 28337925

[43] Fang C, Liu H, Luo K, Yu X. Process and proposal for comprehensive regionalization of Chinese human geography. Journal of Geographical Sciences. 2017;27(10):1155–68. doi: 10.1007/s11442-017-1428-y

[44] Dagum C. A new approach to the decomposition of the Gini income inequality ratio. Empirical Economics. 1997;22(4):515–31. doi: 10.1007/bf01205777

[45] Peng XD, Xu YY, Hu XJ, Guo LJ, Bo J. Analysis of the status and effects of the public-funded free 23-valent pneumococcal polysaccharide vaccination among elderly aged 60 years or older in Shanghai, 2013–2023. Journal of Tuberculosis and Lung Disease. 2023;4:283–94. doi: 10.19983/j.issn.2096-8493.20230064

[46] Sun LK, Li YH, Shi ML. Analysis of influenza vaccination status of primary and secondary school students in Shijingshan District of Beiiing from 2019 to 2023. Chinese Journal of School Health. 2023;44:935–7+41. doi: 10.16835/j.cnki.1000-9817.2023.06.031

[47] Zhang Q, Sun HZ, Lin QY, Lin KM, Chong KM. Public network attention to hiking in China and its influencing factors. Plos One. 2024;19(7):e0306726. doi: 10.1371/journal.pone.0306726 WOS:001272317500111. 38991020 PMC11239110

[48] Guo XJ, Zhang J, Wu XL. Spatio-temporal characteristics of the novel coronavirus attention network and its influencing factors in China. Plos One. 2021;16(9):e0257291. doi: 10.1371/journal.pone.0257291 WOS:000707071500045. 34529727 PMC8445458

[49] Alzghaibi H. People behavioral during health information searching in COVID-19 era: a review. Front Public Health. 2023;11:1166639. doi: 10.3389/fpubh.2023.1166639 WOS:001093673400001. 37637820 PMC10449606

[50] Wong DWS. Issues in the Current Practices of Spatial Cluster Detection and Exploring Alternative Methods. Int J Env Res Pub He. 2021;18(18):9848. doi: 10.3390/ijerph18189848 WOS:000699528100001. 34574771 PMC8466737

[51] Xiao GN, Xiao Y, Shu YQ, Ni AN, Jiang ZR. Technical and economic analysis of battery electric buses with different charging rates. Transport Res D-Tr E. 2024;132:104254. doi: 10.1016/j.trd.2024.104254 WOS:001243663000001.

[52] Fang CL, Yu XH, Zhang XL, Fang JW, Liu HM. Big data analysis on the spatial networks of urban agglomeration. Cities. 2020;102:102735. doi: 10.1016/j.cities.2020.102735 WOS:000534585300002.

[53] Wang F, Fan WN, Lin XY, Liu J, Ye X. Does Population Mobility Contribute to Urbanization Convergence? Empirical Evidence from Three Major Urban Agglomerations in China. Sustainability-Basel. 2020;12(2):458. doi: 10.3390/su12020458 WOS:000516824600016.

[54] Ge FJ, Chen WX, Zeng YY, Li JF. The Nexus between Urbanization and Traffic Accessibility in the Middle Reaches of the Yangtze River Urban Agglomerations, China. Int J Env Res Pub He. 2021;18(7):3828. doi: 10.3390/ijerph18073828 WOS:000638506400001. 33917504 PMC8038807

