# Supplementary material for: Spatiotemporal dynamic and regional differences of public attention to vaccination: An empirical study in China
Source: PLoS One. 2024 Dec 23;19(12):e0312488. doi: 10.1371/journal.pone.0312488 (PMC11666014; doi:10.1371/journal.pone.0312488)
Supplement: S1 Table — (DOCX) [file pone.0312488.s001.docx]

| **Region No.** | **Name of Human Geography Region** | **Administrative divisions included in the scope** |
| --- | --- | --- |
| **Ⅰ** | Northeast China | Heilongjiang, Liaoning, Jilin, eastern Inner Mongolia(Chifeng City, Tongliao City, Hulun Bu City, Hinggan League, Xilin Gol League) |
| **Ⅱ** | North China | Beijing, Tianjin, Hebei, Shanxi, northern and central Shandong, central Inner Mongolia(Hohhot, Baotou City, Wulanchabu City) |
| **Ⅲ** | East China | Shanghai, Zhejiang, Jiangsu, Anhui, southern Shandong(Zaozhuang City) |
| **Ⅳ** | Central China | Hubei, Jiangxi, Hunan, central and eastern Henan, southern Guizhou(Tongren City, Qiandongnan Miao, and Dong Autonomous Prefecture), northern Guangxi(Guilin City) |
| **Ⅴ** | South China | Fujian, Guangdong, Taiwan, Hainan, eastern and western Guangxi,, Hong Kong , Macau |
| **Ⅵ** | Northwest China | Shaanxi, Ningxia, Xinjiang, central and northern Gansu, western Inner Mongolia(Wuhai City, Ordos City, Bayannur City, Alxa League), northeast Qinghai(Xining City, Haidong City), western Henan(Sanmenxia City ) |
| **Ⅶ** | Southwest China | Yunnan, eastern Sichuan, Chongqing, western Guizhou |
| **Ⅷ** | Qinghai-Tibet | Tibet, central and western Qinghai western Sichuan(Aba Tibetan and Qiang Autonomous Prefecture, Ganzi Tibetan Autonomous Prefecture), southern Gansu(Longnan City Gannan Tibetan Autonomous Prefecture) |
